# Femoral stem alignment and post-operative patient satisfaction: a retrospective analysis of total hip arthroplasty using computer-assisted equipment for placing cups

**DOI:** 10.1007/s00264-026-06949-2

**Published:** 2026-07-07

**Authors:** Hiroki Kaneta, Takeshi Shoji, Shinichi Ueki, Hiroyuki Morita, Yosuke Kozuma, Naoto Nakayama, Shuhei Yamasaki, Nobuo Adachi

**Affiliations:** Department of Orthopaedic Surgery, Graduate School of Biomedical and Health Sciences, Hiroshima University, Hiroshima, Japan

**Keywords:** Computer-assisted surgery, Femoral anteversion, Femoral stem alignment, Patient satisfaction, Patient-reported outcome measures, Total hip arthroplasty

## Abstract

**Purpose:**

Using computer-assisted equipment (CAE) in total hip arthroplasty (THA) has facilitated improved accuracy of acetabular cup placement, but the impact of aligning the femoral stem on post-operative patient satisfaction remains unclear. In this study, we evaluated the post-operative patient-reported outcome measures (PROMs) and identified factors related to femoral stem alignment associated with post-operative PROMs after THA.

**Methods:**

This retrospective study included 200 patients (205 hips; mean age, 67.3 ± 9.8 years) undergoing THA using CAE for acetabular cup placement at our hospital. Alignment parameters of the femoral stem, including stem anteversion, change in stem anteversion, femoral offset, and coronal and sagittal stem angles, were evaluated using computed tomography scans and radiographs. Patients were categorised into the normal and Low&High alignment groups based on predefined, clinically acceptable ranges and deviation from native femoral anatomy. Patient satisfaction was assessed using a visual analogue scale and clinical outcomes were evaluated using the Japanese Orthopaedic Association Hip Disease Evaluation Questionnaire (JHEQ) at one year post-operatively.

**Results:**

Patients in the normal alignment group demonstrated significantly higher JHEQ total and movement scores than those in the Low&High group. Through multivariate logistic regression analysis, we identified change in stem anteversion as an independent factor associated with high post-operative patient satisfaction.

**Conclusion:**

Accurate femoral stem alignment, particularly minimizing deviations in stem anteversion from the native femoral anteversion, was significantly associated with improved post-operative PROMs after computer-assisted THA. These results highlight the importance of achieving accurate femoral stem alignment, even when acetabular cup placement is optimised using CAE.

**Supplementary Information:**

The online version contains supplementary material available at https://doi.org/10.1007/s00264-026-06949-2.

## Introduction

Total hip arthroplasty (THA) is an effective surgical treatment for alleviating pain and restoring function in patients with hip disorders such as osteoarthritis, osteonecrosis of the femoral head, and rheumatoid arthritis [1]. Accurately positioning the implant is essential for achieving optimal clinical outcomes because malposition may lead to dislocation, impingement, discrepancy in leg length, prosthetic wear, and early revision surgery [2–4].

Recent advances in computer-assisted equipment (CAE) include robotic-assisted systems, computed tomography (CT)-based navigation, and portable augmented reality-based navigation. Such advances have significantly improved the accuracy of acetabular cup placement during THA by providing real-time intraoperative guidance [5, 6].

However, despite these technological advances and their increasing adoption, improvements in radiographic accuracy of cup placement do not necessarily translate into better post-operative patient-reported outcome measures (PROMs) [7, 8].

Kaneta et al. further demonstrated that, among different CAE modalities, the smallest cup placement errors were achieved with robotic-assisted systems. Yet this increased accuracy did not result in improved post-operative PROMs [9]. Thus, factors beyond acetabular positioning, particularly those related to the femoral component, may influence post-operative satisfaction.

Femoral stem alignment has been reported to play a crucial role in restoring hip biomechanics and ensuring joint stability [10]. Malalignment in stem anteversion, coronal or sagittal alignment, or offset may lead to altered joint kinematics, soft tissue imbalance, or impingement, ultimately affecting patient-perceived outcomes [11, 12]. However, although previous studies have investigated radiological and functional outcomes after THA, only a few have evaluated post-operative PROMs from the perspective of stem alignment and quantitatively examined the association between detailed stem alignment parameters and post-operative PROMs in computer-assisted THA.

Therefore, this study investigated the post-operative PROMs and the association between femoral stem alignment and post-operative PROMs under standardised, computer-assisted acetabular cup placement.

We hypothesised that femoral stem alignment has an impact on post-operative PROMs and that alignment closer to the native femoral anatomy, particularly minimal change in stem anteversion, would be associated with higher patient satisfaction in computer-assisted THA.

## Materials and methods

### Study participants

This retrospective single-institution study adhered to principles of the Declaration of Helsinki and was approved by the institutional review board of our hospital. Informed consent was obtained from all participants.

Patients who underwent primary THA using one of three CAE systems for acetabular cup placement at our hospital between May 2021 and August 2023, with at least 12 months follow-up, were included. The CAE systems included robotic-assisted (Mako; Stryker, Kalamazoo, MI, USA), CT-based navigation (CT-Hip System version 1.1, Stryker, Freiburg, Germany), and portable navigation (AR Hip; Zimmer Biomet Japan, Tokyo, Japan). Patients undergoing bilateral or revision THA, those with neurological, knee, or spinal diseases, contralateral severe hip osteoarthritis, arthroplasty, non-elective or oncological THA, or missing PROM data were excluded [5, 13]. Spinal disease was defined as any case of lumbar spinal stenosis, scoliosis, or vertebral compression fractures identified using whole-spine X-ray examinations, because these may influence PROMs and spino-pelvic mobility. All patients participated in our institution-wide comprehensive THA pathway program, including uniform protocols for perioperative care, institutional post-operative rehabilitation, and pain management.

### Pre-operative planning

For all patients, pre-operative planning for both the acetabular and femoral components was performed using a CT-based three-dimensional template, with the supine functional pelvic plane used as the radiographic reference [14]. To improve reproducibility of the functional pelvic plane during CT scanning, a pillow was placed under the sacrum to align the pelvis in the supine position with the bilateral anterior superior iliac spines in the same horizontal plane. The inter-teardrop line was used as the mediolateral axis.

The target cup inclination was set at 40° and the anteversion angle was determined according to Widmer’s combined anteversion theory, adjusting it to 15° ± 5° based on individual patient anatomy [15]. The planned stem version was calculated to achieve the optimal combined anteversion and avoid impingement.

### Surgical procedure

A single surgeon performed THA in all patients using the anterolateral supine approach. Based on a study by Kaneta et al., one of the three CAE systems was used to assist acetabular cup placement [9]. To mimimise selection bias, the type of CAE system used for each patient was randomly assigned.

A cementless Taperloc Microplasty stem with a G7 acetabular cup (Zimmer Biomet) or an Accolade II femoral stem with a Trident II Tritanium cup (Stryker) was implanted in all patients. These were combined with a highly cross-linked polyethylene liner and a 32- or 36-mm delta ceramic head. The femoral stem was inserted according to the manufacturer’s recommendations, with careful attention given to achieving anatomical alignment in terms of anteversion, varus–valgus angle, flexion–extension angle, offset, and leg length.

### Radiographic evaluation

At 3 months post-operatively, all patients underwent biplanar radiographic imaging of the hip, including a supine anteroposterior pelvic view in the neutral position and a cross-table lateral view [16]. Hip CT scans, extending from the anterior superior iliac spine to the distal femoral condyles through the knee joint, were obtained using a 320-row multidetector helical CT scanner (detector beam collimation: 80 × 0.5 mm^2^, 40 mm; slice thickness: 1.00 mm; slice interval: 1.00 mm; Aquilion ONE; Toshiba Medical Healthcare, Tochigi, Japan) [13].

### Measurement of stem alignment

**Fig. 1:**
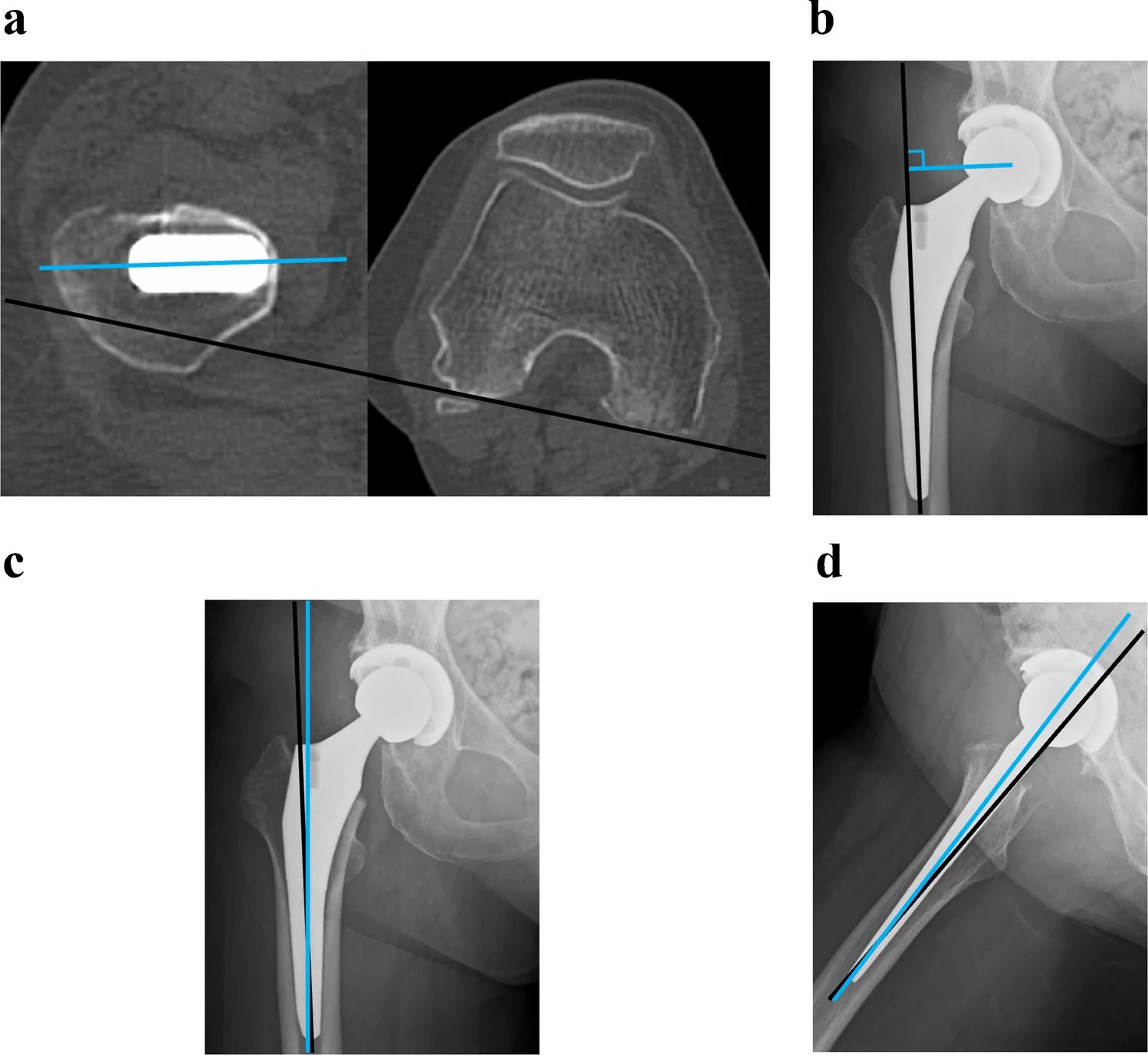
Methods for measuring the alignment parameters of the femoral stem. **a** Stem anteversion was measured using axial computed tomography scans as the angle between the posterior condylar line (black line) and the axis of the stem neck (blue line). **b** Femoral offset was measured as the perpendicular distance from the centre of the femoral head to the axis of the femoral shaft on coronal radiographs; the femoral shaft axis is shown in black and the perpendicular offset distance in blue. **c** The coronal stem angle was measured as the angle between the femoral anatomical axis (black line) and femoral stem axis (blue line) on coronal radiographs. **d** The sagittal stem angle was measured as the angle between the femoral anatomical axis (black line) and femoral stem axis (blue line) using sagittal radiographs

Stem alignment parameters were evaluated according to the recommendations of the International Society of Biomechanics [17]. The anteversion of the femoral stem was assessed using axial CT scans obtained in the supine position at 3 months post-operatively, covering the region from the pelvis to the distal femur [18]. Measurements of femoral offset [19], and coronal and sagittal stem angles [20] were obtained according to the descriptions illustrated in Fig. 1. Measurement reliability was excellent for all stem alignment parameters (Table S1).

Normal ranges were defined for each parameter based on previous studies and the clinically acceptable range of measurement or biomechanical influence previously reported [4, 13, 21–23]: stem anteversion, 10°–40° [13]; change in stem anteversion (post-operative minus pre-operative), ± 5° (positive for increased anteversion, negative for decreased) [23]; femoral offset, difference between the operated and contralateral side ± 3 mm [21]; coronal angle, ± 3° (positive for varus, negative for valgus) [13]; and sagittal angle, ± 3° (positive for flexion, negative for extension) [4]. These thresholds were based on previously published studies and represent clinically accepted ranges used to define appropriate implant alignment in total hip arthroplasty, enabling clinically meaningful group comparisons. For comparison of post-operative outcomes, patients were then classified into the Normal or Low&High groups based on whether the measured values fell within or outside the normal range.

Leg length discrepancy (LLD) was measured on an anteroposterior pelvic radiograph using the vertical distance from the inter-teardrop line to the most prominent aspect of the lesser trochanter on each side, according to a previously described method [24]. LLD was calculated as the difference between the operated and contralateral sides (positive values indicating lengthening and negative values indicating shortening).

### Patient-reported outcomes

Clinical evaluation was conducted using the Japanese Orthopaedic Association Hip Disease Evaluation Questionnaire (JHEQ), a PROM used to assess the clinical condition of patients with hip disorders. The total JHEQ score ranges from 0 (worst) to 84 (best) points and includes three subscales: pain (0–28 points), movement (0–28 points), and mental (0–28 points). For each subscale, 0 points represents the worst outcome and 28 points represents the best [25]. Overall patient satisfaction was assessed using a visual analogue scale (VAS) for satisfaction, with 0 points representing the worst possible satisfaction and 100 points representing the best [26]. The JHEQ and VAS satisfaction were administered pre-operatively and at one year post-operatively.

### Statistical analysis

We used the Mann–Whitney U test to compare each parameter between the two stem-alignment groups. For stem anteversion change, stratified into three groups, we used the Kruskal–Wallis followed by Dunn–Bonferroni post hoc tests. Categorical variables were compared using the chi-squared test. We performed logistic regression analysis to identify independent stem alignment factors associated with higher post-operative patient satisfaction scores assessed using the VAS satisfaction. Patient satisfaction was treated as a binary outcome, with a VAS score of ≥ 75 defined as satisfied (1) and a score of < 75 defined as unsatisfied (0), according to a previous study [27]. Odds ratios with 95% confidence intervals were calculated to estimate the strength of the association between stem alignment parameters and patient satisfaction. The explanatory variables included stem anteversion, change in stem anteversion, femoral offset difference, coronal and sagittal stem angles, and LLD to adjust for the potential influence of limb length restoration on patient satisfaction. Continuous variables are presented as mean ± standard deviation (range). Statistical significance was set at *p* < 0.05. Statistical analyses were performed using GraphPad Prism 10.0 (San Diego, CA, USA).

## Results

**Fig. 2:**
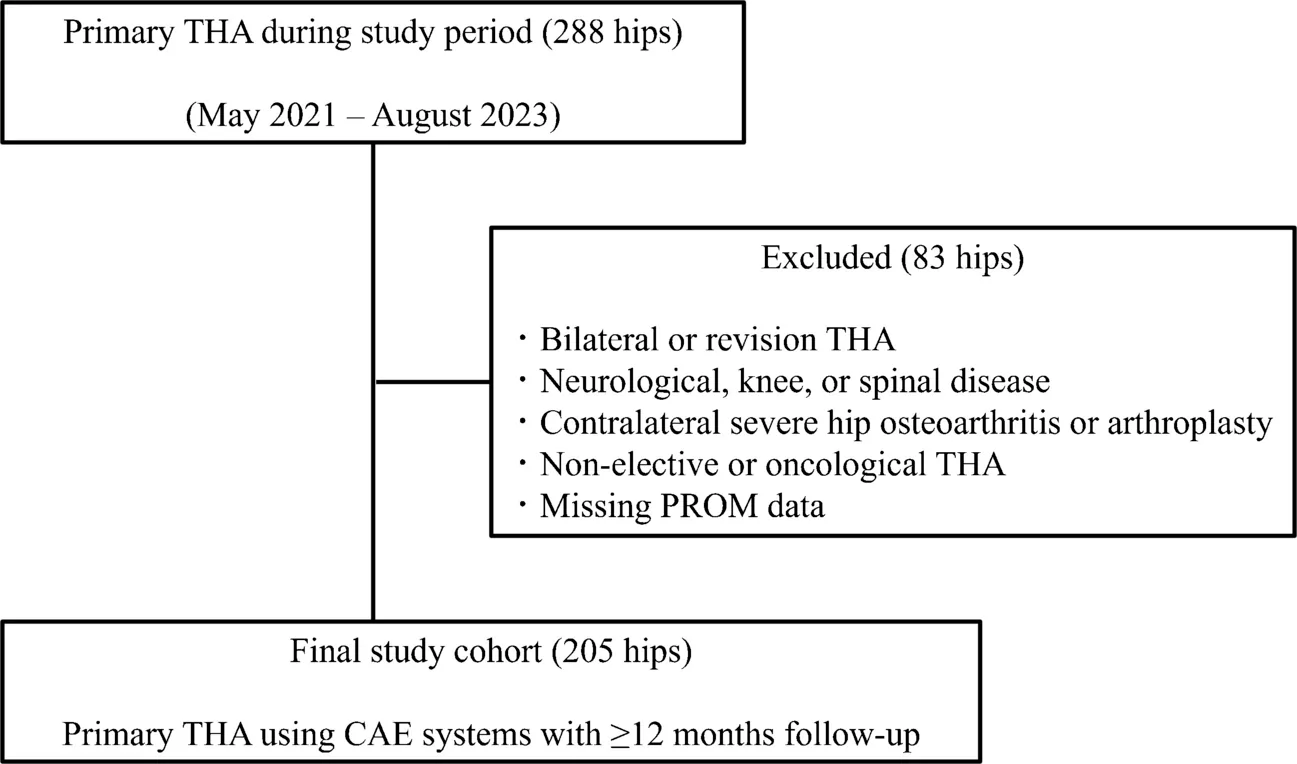
Flowchart of patient screening and inclusion in the study. THA, total hip arthroplasty; PROMs, patient-reported outcome measures; CAE, computer-assisted equipment

Two hundred patients (205 hips) underwent primary THA using one of three CAE systems for cup placement (Fig. 2). Patient demographics, complications, and stem alignment parameters are summarised in Table 1. The mean age was 67.3 ± 9.8 years; 45 male and 160 female hips. The primary diagnoses were osteoarthritis (165 hips) and osteonecrosis of the femoral head (40 hips). The average change in stem anteversion was + 1.5° ± 6.7°, indicating a slight post-operative increase. The distribution of changes in stem anteversion is shown in Supplementary Fig. S1. Other stem alignment parameters are summarized in Table 1. No post-operative complications, including dislocation, infection, fracture, readmission, or revision surgery were observed.

**Table 1:** Characteristics of patients and clinical outcomes

Variable	Categorisation/Unit of measure	Value
Patient	(hips)	205
Age	(years)	67.3 ± 9.8 (44 to 88)
Sex	(Male/Female) (n)	45/160
Primary disease	(OA/ON) (n)	165/40
Body mass index	(kg/m^2^)	23.3 ± 3.5 (16.7 to 30.3)
Post-operative complications		
Dislocation	(n)	0
Infection	(n)	0
Fracture	(n)	0
Readmission	(n)	0
Revision	(n)	0
Radiographic parameters		
Stem anteversion	(°)	23.0 ± 10.7 (2.0 to 56.6)
Change in stem anteversion	(°)	1.5 ± 6.7 (−19.5 to 22.3)
Femoral offset	(mm)	1.1 ± 3.1 (−6.8 to 9.5)
Coronal angle	(°)	0.7 ± 1.5 (−5.0 to 4.5)
Sagittal angle	(°)	2.8 ± 1.9 (−4.5 to 7.8)
Leg length discrepancy	(mm)	0.0 ± 4.3 (−6.8 to 6.2)

Stem anteversion: Angle between the stem neck axis and posterior femoral condylar line on axial computed tomography scans (°)

Change in stem anteversion: Post-operative minus pre-operative stem anteversion (°)

Femoral offset: Perpendicular distance from the centre of the femoral head to the femoral shaft axis, expressed as the difference between the operated and contralateral sides (mm)

Coronal stem angle: Angle between the femoral stem axis and femoral anatomical axis on coronal radiographs (°)

Sagittal stem angle: Angle between the femoral stem axis and femoral anatomical axis on sagittal radiographs (°)

Leg length discrepancy: Difference in the vertical distance from the inter-teardrop line to the most prominent aspect of the lesser trochanter between the operated and contralateral sides on anteroposterior pelvic radiographs (mm)

Data are shown as mean ± standard deviation (range)

OA, osteoarthritis; ON, osteonecrosis of the femoral head

PROMs significantly improved after surgery (Table S2). Patient satisfaction (VAS) increased from 19.2 ± 17.0 points pre-operatively to 90.7 ± 14.9 points at 1 year post-operatively. The total JHEQ score also improved significantly from before to after surgery, with all subscales also showing significant post-operative improvements.

Baseline characteristics of Normal vs Low&High groups showed no significant differences in age, sex, body mass index, or primary diagnosis for any alignment parameter (Table 2).

**Table 2:** Characteristics of patients according to stem alignment

Variable	Categorisation/Unit of measure	Normal group	Low&High group	*p-*Value
Stem anteversion				
Patient	(hips)	176	29	
Age	(years)	67.3 ± 9.8 (44 to 88)	67.5 ± 9.4 (44 to 88)	0.908
Sex	(Male/Female) (n)	35/141	10/19	0.149
Primary disease	(OA/ON) (n)	144/32	21/8	0.391
Body mass index	(kg/m^2^)	23.2 ± 3.2 (16.7 to 30.3)	24.0 ± 5.1 (18.2 to 30.1)	0.840
Change in stem anteversion				
Patient	(hips)	133	72	
Age	(years)	66.1 ± 9.6 (44 to 88)	69.5 ± 9.8 (44 to 88)	0.069
Sex	(Male/Female) (n)	28/105	17/55	0.590
Primary disease	(OA/ON) (n)	109/24	56/16	0.454
Body mass index	(kg/m^2^)	23.0 ± 2.9 (18.2 to 30.3)	22.9 ± 3.7 (16.7 to 29.6)	0.716
Femoral offset				
Patient	(hips)	155	50	
Age	(years)	67.9 ± 10.0 (44 to 88)	65.5 ± 8.8 (45 to 85)	0.147
Sex	(Male/Female) (n)	40/115	5/45	0.079
Primary disease	(OA/ON) (n)	122/33	43/7	0.396
Body mass index	(kg/m^2^)	23.4 ± 3.5 (16.7 to 30.3)	22.9 ± 3.5 (18.4 to 28.5)	0.706
Coronal angle				
Patient	(hips)	190	15	
Age	(years)	67.1 ± 9.7 (44 to 88)	70.1 ± 10.8 (51 to 85)	0.256
Sex	(Male/Female) (n)	39/151	6/9	0.140
Primary disease	(OA/ON) (n)	152/38	13/2	0.788
Body mass index	(kg/m^2^)	23.9 ± 3.3 (16.7 to 30.3)	26.8 ± 2.8 (24.8 to 30.3)	0.059
Sagittal angle				
Patient	(hips)	130	75	
Age	(years)	68.0 ± 9.2 (45 to 87)	66.2 ± 10.6 (44 to 88)	0.247
Sex	(Male/Female) (n)	26/104	19/56	0.414
Primary disease	(OA/ON) (n)	106/24	59/16	0.597
Body mass index	(kg/m^2^)	23.2 ± 3.6 (16.7 to 30.3)	23.6 ± 3.3 (18.2 to 29.6)	0.662

Stem anteversion: Angle between the stem neck axis and the posterior femoral condylar line on axial compute tomography scans (°)

Change in stem anteversion: Post-operative minus pre-operative stem anteversion (°)

Femoral offset: Perpendicular distance from the centre of the femoral head to the femoral shaft axis, expressed as the difference between the operated and contralateral sides (mm)

Coronal stem angle: Angle between the femoral stem axis and femoral anatomical axis on coronal radiographs (°)

Sagittal stem angle: Angle between the femoral stem axis and femoral anatomical axis on sagittal radiographs (°)

Definitions for the Normal group: Stem anteversion, 10°–40°; change in stem anteversion, − 5° to + 5°; femoral offset difference, − 3 to + 3 mm; coronal stem angle, − 3° to + 3° (positive = varus); sagittal stem angle, − 3° to + 3° (positive = flexion)

The Low&High group included patients with values below or above the defined normal range

Data are shown as mean ± standard deviation (range)

OA, osteoarthritis; ON, osteonecrosis of the femoral head

Comparisons between the Normal and Low&High groups for each stem alignment parameter are presented in Table 3. All evaluated PROMs for stem anteversion and change in stem anteversion were significantly higher in the Normal group than in the Low&High group (all *p* < 0.05). For femoral offset, no significant between-group differences were observed for any PROMs. Regarding the coronal stem angle, patient satisfaction, total JHEQ score, and movement scores were significantly higher in the Normal group. For the sagittal stem angle, all PROMs were significantly higher in the Normal group (all *p* < 0.05). As an exploratory analysis, post hoc power analysis was conducted based on the observed differences in the patient VAS satisfaction scores between the Normal and Low&High groups for change in stem anteversion and sagittal stem angle. The mean VAS scores were 93.8 ± 12.2 points versus 84.8 ± 17.7 for stem anteversion change, and 93.6 ± 13.1 points versus 85.2 ± 16.7 points for sagittal stem angle. Using these values and the respective sample sizes, the calculated effect sizes were *f* = 0.314 for stem anteversion change and *f* = 0.289 for sagittal stem angle, yielding post hoc statistical powers of 0.970 and 0.951, respectively. These results indicate that the study had sufficient power to detect significant differences in patient satisfaction for these parameters.

**Table 3:** Patient-reported outcome measures according to stem alignment

Scale according to parameter	Unit of measure	Normal group	Low&High group	*p-*Value
Stem anteversion				
Patient satisfaction (VAS) score	(pts)	91.5 ± 14.6 (70 to 100)	86.3 ± 15.8 (50 to 100)	0.037
Total JHEQ score	(pts)	69.9 ± 12.2 (50 to 84)	58.2 ± 11.9 (46 to 82)	< 0.001
Pain score	(pts)	25.5 ± 3.8 (22 to 28)	23.2 ± 4.8 (20 to 28)	0.016
Movement score	(pts)	20.5 ± 5.9 (11 to 28)	14.8 ± 6.3 (4 to 26)	< 0.001
Mental score	(pts)	23.9 ± 5.1 (13 to 28)	20.2 ± 5.8 (11 to 28)	0.001
Change in stem anteversion				
Patient satisfaction (VAS) score	(pts)	93.8 ± 12.2 (70 to 100)	84.8 ± 17.7 (50 to 100)	< 0.001
Total JHEQ score	(pts)	73.1 ± 10.9 (51 to 84)	58.8 ± 11.0 (46 to 84)	< 0.001
Pain score	(pts)	26.4 ± 3.2 (22 to 28)	22.8 ± 4.4 (20 to 28)	< 0.001
Movement score	(pts)	21.6 ± 5.7 (12 to 28)	16.1 ± 5.8 (4 to 28)	< 0.001
Mental score	(pts)	25.1 ± 4.2 (14 to 28)	20.0 ± 5.6 (11 to 28)	< 0.001
Femoral offset				
Patient satisfaction (VAS) score	(pts)	91.2 ± 14.5 (60 to 100)	89.3 ± 16.1 (50 to 100)	0.510
Total JHEQ score	(pts)	68.4 ± 13.1 (52 to 84)	67.5 ± 12.0 (46 to 84)	0.471
Pain score	(pts)	25.2 ± 4.1 (20 to 28)	25.1 ± 3.6 (21 to 28)	0.555
Movement score	(pts)	19.6 ± 6.6 (12 to 28)	19.8 ± 5.4 (4 to 28)	0.813
Mental score	(pts)	23.6 ± 5.3 (13 to 28)	22.6 ± 5.4 (11 to 28)	0.189
Coronal angle				
Patient satisfaction (VAS) score	(pts)	91.5 ± 14.3 (60 to 100)	81.5 ± 19.5 (50 to 100)	0.017
Total JHEQ score	(pts)	69.0 ± 12.5 (51 to 84)	58.6 ± 13.2 (46 to 77)	0.005
Pain score	(pts)	25.3 ± 3.8 (21 to 28)	23.4 ± 5.8 (20 to 28)	0.173
Movement score	(pts)	20.1 ± 6.1 (12 to 28)	14.5 ± 6.7 (4 to 23)	0.003
Mental score	(pts)	23.6 ± 5.1 (11 to 28)	20.8 ± 6.9 (11 to 28)	0.126
Sagittal angle				
Patient satisfaction (VAS) score	(pts)	93.6 ± 13.1 (70 to 100)	85.2 ± 16.7 (50 to 100)	< 0.001
Total JHEQ score	(pts)	72.5 ± 10.2 (54 to 84)	61.2 ± 13.7 (46 to 84)	< 0.001
Pain score	(pts)	26.2 ± 3.0 (22 to 28)	23.5 ± 4.8 (20 to 28)	< 0.001
Movement score	(pts)	21.1 ± 5.7 (12 to 28)	17.3 ± 6.5 (4 to 28)	< 0.001
Mental score	(pts)	25.1 ± 4.0 (14 to 28)	20.4 ± 5.9 (11 to 28)	< 0.001

Stem anteversion: Angle between the stem neck axis and the posterior femoral condylar line on axial computed tomography scans (°)

Change in stem anteversion: Post-operative minus pre-operative stem anteversion (°)

Femoral offset: Perpendicular distance from the centre of the femoral head to the femoral shaft axis, expressed as the difference between the operated and contralateral sides (mm)

Coronal stem angle: Angle between the femoral stem axis and femoral anatomical axis on coronal radiographs (°)

Sagittal stem angle: Angle between the femoral stem axis and femoral anatomical axis on sagittal radiographs (°)

Normal group definitions: Stem anteversion, 10°–40°; change in stem anteversion, − 5° to + 5°; femoral offset difference, − 3 to + 3 mm; coronal stem angle, − 3° to + 3° (positive = varus); sagittal stem angle, − 3° to + 3° (positive = flexion)

The Low&High group included patients with values below or above the defined normal range

Data are shown as mean ± standard deviation (range)

JHEQ, Japanese Orthopaedic Association Hip Disease Evaluation Questionnaire; pts, points; VAS, visual analogue scale

Using multivariate logistic regression analysis, change in stem anteversion and sagittal stem angle were identified as independent factors positively associated with patient satisfaction (Table 4), whereas LLD was not significantly associated with postoperative patient satisfaction (p = 0.973). The model showed good fit (Hosmer–Lemeshow test, *p* = 0.618) and discrimination (receiver operating characteristic curve analysis, area under the receiver operating characteristic curve = 0.793;* p* < 0.001), indicating good predictive performance.

**Table 4:** Multivariate logistic regression analysis of factors associated with patient satisfaction

Factor	Odds ratio	95% CI	*p*-Value
Stem anteversion	0.24	0.03—1.17	0.080
Change in stem anteversion	8.43	2.72—32.26	< 0.001
Femoral offset	0.87	0.24—2.85	0.825
Coronal angle	0.85	0.04—7.14	0.892
Sagittal angle	3.36	1.11—10.81	0.032
Leg length discrepancy	1.00	0.87–1.15	0.973

Stem anteversion: Angle between the stem neck axis and posterior femoral condylar line on axial computed tomography scans (°)

Change in stem anteversion: Post-operative minus pre-operative stem anteversion (°)

Femoral offset: Perpendicular distance from the centre of the femoral head to the femoral shaft axis, expressed as the difference between the operated and contralateral sides (mm)

Coronal stem angle: Angle between the femoral stem axis and femoral anatomical axis on coronal radiographs (°)

Sagittal stem angle: Angle between the femoral stem axis and femoral anatomical axis on sagittal radiographs (°)

Leg length discrepancy: Difference in the vertical distance from the inter-teardrop line to the most prominent aspect of the lesser trochanter between the operated and contralateral sides on anteroposterior pelvic radiographs (mm)

Patient satisfaction was assessed using a visual analogue scale and treated as a binary outcome (satisfied = 1, unsatisfied = 0)

*CI* confidence interval

Exploratory analysis stratified patients by native femoral anteversion; post-operative changes in stem anteversion were further categorised. Across all pre-operative anteversion groups, patients with minimal changes in stem anteversion demonstrated significantly higher PROMs (Table S3).

## Discussion

We investigated the association between femoral stem alignment and post-operative patient satisfaction after THA performed using CAE for accurate acetabular cup placement. The main findings were that (1) smaller changes in stem anteversion from pre- to post-operative measurements and (2) appropriate sagittal stem alignment were significantly associated with higher patient satisfaction and better PROMs. Using multivariate analysis, we identified these two parameters as independent predictors of patient satisfaction. These results highlight the importance of achieving accurate femoral stem alignment, even when acetabular cup placement is optimised using CAE.

Previous studies have primarily focused on the acetabular component, demonstrating that accurate cup placement reduces complications such as dislocation and impingement [2, 3]. However, Shoji et al. reported that implant-related factors beyond cup positioning influence PROMs [13]. Our observation that patient satisfaction was more strongly related to femoral stem alignment than to other perioperative factors supports this view. Our findings further demonstrated that the change in stem anteversion from pre- to post-operative measurement, rather than the absolute anteversion value alone, was a particularly important determinant of post-operative satisfaction. This finding is consistent with previous reports indicating that native femoral version is predictive of achieved stem version in cementless THA, highlighting the importance of preserving native femoral anatomy when possible [28].

From a biomechanical perspective, excessive deviation of stem anteversion may alter the combined anteversion of the hip joint, potentially leading to impingement or instability [29, 30]. However, recent studies have shown that dislocation may still occur even when target combined anteversion is achieved, indicating that patient-specific femoral morphology and stem alignment remain important considerations [31]. Similarly, inappropriate sagittal stem alignment may affect soft tissue tension, hip flexion mechanics, and load transfer through the femur, possibly contributing to patient discomfort or reduced satisfaction [4, 13]. Our findings suggest that maintaining stem anteversion close to the native femoral version and minimising sagittal malalignment are crucial for restoring normal joint kinematics and achieving favourable PROMs. However, accurately controlling stem version in cementless THA remains challenging, as stem orientation may be influenced by implant design and patient-specific femoral morphology [32]. Within the context of our findings, maintaining stem anteversion within ± 5° of the native femoral version and avoiding sagittal malalignment beyond ± 3° may represent practical alignment targets for optimising early patient satisfaction.

Femoral offset plays an important role in improving abductor muscle function and stabilising gait [33]. However, we found no significant between-group differences in PROMs according to femoral offset. Similarly, LLD was not independently associated with patient satisfaction after adjustment for stem alignment parameters. One possible reason for these consistent findings is that all procedures were performed using the anterolateral supine approach, in which fluoroscopy is used intra-operatively to optimise both femoral offset and LLD [34]. In addition, the use of CAE and fluoroscopic guidance may have reduced variability in post-operative LLD across patients, thereby limiting the presence of clinically meaningful discrepancies large enough to significantly influence PROMs [35].

Although we found no significant associations between femoral offset and early post-operative PROMs, long-term consequences of initial component alignment, including the accuracy of cup placement and orientation of the femoral stem (anteversion, sagittal angle, and offset), could still substantially influence joint function and the risk of revision, effects not captured by early PROMs [36].

Therefore, long-term follow-up studies focusing on stem alignment are warranted. PROMs may also be influenced by factors such as post-operative rehabilitation and patient expectations [37–39].

In this study, CAE was used to assist with placing the acetabular cup to ensure consistent positioning; however, no navigation or robotic systems were used to implant the femoral stem. Despite the use of CAE for acetabular cup placement, femoral stem positioning remained dependent on surgical technique and femoral anatomy. Future studies should focus on pre-operative prediction of stem alignment deviation and on surgical strategies to achieve stem orientation within an optimal range. Considering that the stem alignment parameters were significant predictors of patient satisfaction, future advancements in robotic-assisted THA systems, such as the Mako platform, may help improve the accuracy of both cup and stem alignment [40]. This may further enhance patient-specific hip reconstruction and post-operative satisfaction.

This study had several limitations. First, it was a single-centre, retrospective study, which may have limited the external validity of the findings and introduced the potential for selection bias. Second, all procedures were performed by experienced surgeons via the anterolateral supine approach, and patients with spinal disease were excluded to minimise the influence of spino-pelvic imbalance on PROMs. Therefore, the generalisability of our findings to less experienced surgeons, different surgical approaches, or patients with hip–spine syndrome may be limited [41, 42]. Third, detailed intra-operative data, such as subtle adjustments during stem trialling or variations in bone quality, were unavailable; this may have influenced post-operative outcomes. In addition, stem anteversion was measured using two-dimensional CT slices rather than three-dimensional reconstruction techniques, which may be influenced by scanner settings and measurement methodology [43]. Finally, the follow-up period was limited to early post-operative outcomes, and the one-year results may not reflect long-term mechanical stability or late complications such as stress shielding or implant loosening related to stem alignment [44, 45]. Despite these limitations, the findings provide valuable insights into the association between femoral stem alignment and early PROMs.

In conclusion, in this study, we demonstrated that a minimal change in stem anteversion and appropriate sagittal stem alignment were independently associated with greater post-operative patient satisfaction following THA. These findings emphasise the importance of precise femoral alignment to complement accurate acetabular placement, suggesting that future technologies enabling improved control of stem positioning may further enhance patient-reported outcomes after THA.

## Supplementary Information

Below is the link to the electronic supplementary material.

Supplementary file1 (DOCX 53 KB)

## Data Availability

The datasets during and/or analyzed during the current study available from the corresponding author on reasonable request.
