# Supplementary material for: Femoral stem alignment and post-operative patient satisfaction: a retrospective analysis of total hip arthroplasty using computer-assisted equipment for placing cups
Source: Int Orthop. 2026 Jul 7;50(10):2559–69. doi: 10.1007/s00264-026-06949-2 (PMC13619799; doi:10.1007/s00264-026-06949-2)
Supplement: Supplementary file 1 [file 264_2026_6949_MOESM1_ESM.docx]

**Supplemental Materials and Methods**

**Statistical Analysis**

To investigate the influence of native femoral anatomy on post-operative outcomes, patients were stratified according to pre-operative femoral anteversion into three groups: low (<10°), normal (10°–30°), and high (>30°), based on a previous report on native femoral anteversion distribution in adults [1]. Within each pre-operative anteversion group, post-operative changes in stem anteversion were categorised as low (< −5°), normal (−5° to +5°), or high (> +5°). PROMs were compared among these subgroups.

**Supplemental Tables**

Table S1. Intra- and inter-observer reliability of stem alignment measurements

| Parameter | Unit of measure | Intra-observer ICC | Inter-observer ICC |
| --- | --- | --- | --- |
| Stem anteversion | (°) | 0.945 | 0.928 |
| Femoral offset | (mm) | 0.931 | 0.917 |
| Coronal stem angle | (°) | 0.939 | 0.925 |
| Sagital stem angle | (°) | 0.942 | 0.933 |

Measurements were independently conducted by two experienced observers. ICC values indicate excellent intra- and inter-observer reliability for all parameters.

ICC, intraclass correlation coefficient.

Table S2. Patient-reported outcome measures, assessed pre-operatively and at 1 year post-operatively

| Scale | Unit of measure | Minimum–Maximum score | Pre-operative | Post-operative, at 1 year | *p-*Value |
| --- | --- | --- | --- | --- | --- |
| Patient satisfaction (VAS) score | (pts) | 0-100 | 19.2±17.0 (0 to 50) | 90.7±14.9 (50 to 100) | <0.001 |
| Total JHEQ score | (pts) | 0-84 | 22.5±12.0 (0 to 60) | 68.2±12.8 (46 to 84) | <0.001 |
| Pain score | (pts) | 0-28 | 7.8±5.3 (0 to 22) | 25.2±4.0 (20 to 28) | <0.001 |
| Movement score | (pts) | 0-28 | 5.3±4.5 (0 to 21) | 19.7±6.3 (4 to 28) | <0.001 |
| Mental score | (pts) | 0-28 | 9.2±5.6 (0 to 26) | 23.3±5.3 (11 to 28) | <0.001 |

Data are shown as mean ± standard deviation (range).

JHEQ, Japanese Orthopaedic Association Hip Disease Evaluation Questionnaire; pts, points; VAS, visual analogue scale.

Table S3. Patient-reported outcome measures according to pre-operative femoral anteversion and post-operative change in stem anteversion

| Pre-operative femoral anteversion | Post-operative change in stem anteversion | Patients (n) | Total JHEQ score (pts) | Pain score (pts) | Movement score (pts) | Mental score (pts) |
| --- | --- | --- | --- | --- | --- | --- |
| Low (<10°) | Low (< −5°) | 11 | 67.6±20.5 (46 to 84) | 24.6±5.0 (20 to 28) | 19.5±10.6 (4 to 28) | 22.3±6.4 (13 to 28) |
|  | Normal (−5° to +5°) | 24 | 75.6±8.0 (56 to 84) ^d^ | 27.6±1.6 (22 to 28) ^d^ | 22.8±5.0 (15 to 28) ^c^ | 25.2±3.6 (16 to 28) ^c^ |
|  | High (> +5°) | 25 | 59.8±11.0 (46 to 84) | 23.3±3.9 (20 to 28) | 16.9±5.3 (8 to 28) | 19.7±6.1 (11 to 28) |
|  |  |  |  |  |  |  |
| Normal (10°–30°) | Low (< −5°) | 14 | 57.0±6.9 (48 to 67) | 22.7±3.2 (20 to 28) | 13.9±6.2 (4 to 22) | 20.4±5.6 (11 to 28) |
|  | Normal (−5° to +5°) | 64 | 75.3±9.1 (52 to 84) ^b, d^ | 26.7±2.7 (22 to 28) ^b^ | 23.2±4.4 (14 to 28) ^b, c^ | 25.4±3.9 (13 to 28) ^a, d^ |
|  | High (> +5°) | 17 | 60.8±13.8 (46 to 84) | 23.6±4.3 (20 to 28) | 18.0±5.9 (9 to 28) | 19.3±6.2 (11 to 28) |
|  |  |  |  |  |  |  |
| High (>30°) | Low (< −5°) | 15 | 63.8±10.0 (53 to 79) | 22.0±7.1 (20 to 28) | 18.5±4.3 (13 to 24) | 23.3±4.1 (19 to 28) |
|  | Normal (−5° to +5°) | 24 | 72.6±11.2 (54 to 84) ^c^ | 26.5±2.3 (22 to 28) | 20.8±6.5 (12 to 28) ^c^ | 25.2±3.9 (17 to 28) |
|  | High (> +5°) | 11 | 57.0±8.6 (46 to 67) | 25.8±2.6 (23 to 28) | 11.8±5.9 (4 to 20) | 19.5±5.7 (11 to 23) |

Data are shown as mean ± standard deviation (range).

^a^ Versus Low group, *p* < 0.05.

^b^ Versus Low group, *p* < 0.01.

^c^ Versus High group, *p* < 0.05.

^d^ Versus High group, *p* < 0.01.

JHEQ, Japanese Orthopaedic Association Hip Disease Evaluation Questionnaire; pts, points.

**Supplemental Figure**

Fig. S1 Distribution of changes in stem anteversion following total hip arthroplasty

Histogram showing the distribution of changes in stem anteversion from pre-operative native femoral version to post-operative stem version in 205 hips following total hip arthroplasty. Dotted lines indicate the predefined normal range (±5°).

**Supplemental References**

1.	Pierrepont JW, Marel E, Bare JV, Walter LR, Stambouzou CZ, Solomon MI, McMahon S, Shimmin AJ (2020) Variation in femoral anteversion in patients requiring total hip replacement. Hip Int 30(3):281-287. <https://doi.org/10.1177/1120700019848088>
